# Measuring food provision in Western Australian long day care (LDC) services: a weighed food record method/protocol at a service level

**DOI:** 10.1186/s12937-019-0462-2

**Published:** 2019-07-16

**Authors:** Ros Sambell, Ruth Wallace, Leesa Costello, Johnny Lo, Amanda Devine

**Affiliations:** Edith Cowan University, 270 Joondalup Drive, Joondalup, WA 6027 Australia

**Keywords:** Long daycare, Food provision, Measurement, Study protocol, Childcare, Nutrition

## Abstract

**Background:**

There are currently 1.3 million children utilising Early Childhood Education and Care (ECEC) services in Australia. Long day care (LDC), family day care and out of school hours care currently provide this service in different environments. This research reports findings from a LDC perspective. Children can consume 40–67% of their food intake whilst at LDC services, this highlights the importance of monitoring food provision at a service level. There are several methods to measure food provision which typically focus on intake at an individual level. There is limited evidence of measuring food provision accurately at a service level and for young children. Accurate and consistent dietary assessment methods are required to determine compliance with dietary guidelines and to provide rigour for comparison between studies.

**Methods:**

Convenience sampling was used to recruit 30 LDC services and food provision assessed over two consecutive days. To ensure consistency, trained researchers weighed raw food ingredients used in food preparation at each service. Food and food weights were allocated to food groups to determine average serves of food group provision at morning tea, lunch and afternoon tea per child. All data were entered into Foodworks for dietary analysis and compliance with dietary guidelines was assessed using Wilcoxon signed-rank and one-sample t-tests (SPSS).

**Discussion:**

This paper outlines the process of data collection for the measurement and auditing of food provision and food waste at a service level. There is an urgent need to ensure that food provision at a service level complies with current dietary guidelines and is accurately assessed. Following a standard method of data collection will allow a more accurate comparison between studies and allow change to be monitored more accurately over time to guide decision makers.

**Trial registration:**

As this research project is conducted at a service level and not a clinical trial, registration was not required.

## Introduction

In 2018, 1,316,350 children attended Early Childhood Education and Care (ECEC) services in Australia [1]. This figure includes children who attended Long Day Care (LDC), Family Day Care and Outside of School Hours Care. In 2018, 751,450 children were in LDC and, on average, spent 25.7 h per week in care [1]. LDC has the highest proportion of children (57.2%) compared to other service types [1]. Typically, these are services that provide children with education and care for more than 8 h a day, 5 days a week and include meals (specifically morning tea (MT), lunch (L) and afternoon tea (AT)) prepared on site. Thirty seven percent of all children aged 2–3 years attended formal care which was most likely to be LDC [2]. Nationally, demand for formal care exceeds supply; currently, the deficit is approximately 9% [2]. Hence, there is a pressing need to grow this sector which also makes good economic sense, amounting to a 3.9% increase valued at $12 billion per year [3].

The ECEC sector provides education and support for children at a rapid stage of growth and development; which coincides with the formation of dietary habits [4–6]. Establishing healthy dietary habits at an early age that support healthy food preferences, has been shown to continue into adulthood and reduce the risk of obesity and non-communicable disease [7–10]. Given this critical time point and the number of children being provided food in LDC, there is an urgent need to ensure that food provision at a service level complies with current dietary guidelines and is accurately assessed.

There have been various intervention strategies implemented in past decades to improve food provision in LDC which have involved face to face training and award schemes [7, 8, 11–14]. The Australian Dietary Guidelines (ADG) have based recommended intakes on core food groups primarily because people eat food not nutrients. These guidelines also provide recommendations for children under 4 years [15]. More recently, interventions have been based on encouraging compliance with the current ADG (2013) [16, 17], which recommend compliance in provision of food group serves for children from 6 months of age. To monitor compliance with dietary recommendations, food group provision needs to be measured at the service level to ensure that, on average, children are being provided with the correct number of servings of food groups and that there is an opportunity to consume the recommended serves of food groups appropriate for the child’s age and gender.

Research suggests that children attending LDC should consume at least 50% of their daily food intake during morning tea (MT), lunch (L) and afternoon tea (AT) [18–21] and reports have indicated that children consume between 40 and 67% of their food intake whilst attending LDC services [11, 22, 23]. This makes LDC settings ideal venues to ensure dietary guidelines are being met and to positively influence the nutritional status of children [24–27].

There is, however, limited evidence of whether food provision in LDC meets current dietary guidelines in Australia. Assumptions of inadequate food provision are typically based on limited reviews of menus [28, 29], food budgets [28], observation methodology [30, 31] rather than “actual” provision, determined from measuring ingredient weights across a range of meals and days at a service [28, 32, 33]. The underpinning premise of measuring food provision is based on the understanding that if core food groups are underprovided (compared to dietary guideline food group serve recommendations), then food groups, most likely, will be “under consumed”. Hence it is unlikely that the recommended food group serves would be met by an individual child, jeopardising health and developmental benefits from optimal nutrition.

Historically, Australian government and non-government organisations have prioritised nutrition in LDC, yet the limited and varied evidence available from the last two decades suggests little improvement in compliance with dietary guidelines or serve recommendations [22, 34, 35]. In addition, the change of serve size and recommended number of serves between 2003 and 2013 [35, 36], the lack of recommended procedures for monitoring food provision, and limited research, makes comparison of research from different studies over time difficult and less likely to provide an accurate representation of trends and the current status [37].

It is generally recognised that the “gold standard” for determining individual food intake is a weighed food record [37–39]. At a service level, there is currently limited evidence of accurate estimates of food provision nor is there a recommended “gold standard” [8, 40]. This protocol paper intends to bridge this gap by contributing knowledge towards the development of a gold standard measure. Specifically, it provides a data collection method used to measure food group provision and food waste in LDC services based on weighed food record at a service level. This will be useful for researchers, industry and resource development by providing evidence to help understand food provision at a service level, monitor change across time and allow for comparative studies based on a common methodology.

### Evidence of variation of data collection methods

Research has determined that food provided in the ECEC sector from Australia [34], USA [41–43] and the Netherlands [44, 45] does not generally meet respective nutritional requirements for children based on dietary recommendations for the individual. Moreover, previous researchers have used different methodologies to determine the adequacy of food provision. These include: individual food records to determine foods and/or nutrient provision or intake [33, 45, 46]; food provision assessment by menu review [28, 29, 47]; food intake by survey or self-reporting [26, 48, 49]; and plate wastage [17].

In addition, the methods to determine food provision vary between studies [13, 50–52]. From this perspective, the issues are two-fold: comparison between research findings cannot be truly accurate if different data collection methods were used; and that these methodologies, such as, menu reviews, observations, surveys, may not provide a true reflection of the actual food provided, which limits the ability of these studies to accurately assess food provision [30, 31]. Further evidence of variation in data collection methods and limitations are summarised below.

### Observation method

Erinosho et al. [53] used the Healthy Eating Index-2005 (HEI-2005) to evaluate foods offered in 20 LDC in North Carolina, United States. Food serves were recorded by an observation method to determine individual dietary intake. Direct observation of dietary intake is often a means of validating a dietary assessment method. Trained observers note down, but not limited to, eating behaviours, food ingredients and portions during a nominated period. This method is not considered a suitable level to determining a habitual dietary pattern at an individual or group level [54]. The total mean score of food and beverages provided to children in day care services was significantly lower than the optimal recommended score (59.12 vs optimal score 100; *P* < 0.01). Despite their belief that observation was a suitable method to determine individual food intake, the 8 year delay in data analysis from 2005 to 2013 is a major limitation as child care practices may have altered. In a further study, Padget and Briley [55] used observation combined with weight estimation with measuring cups as a method to determine individual food consumption. The researchers also considered this a suitable method despite commentary regarding increasing the days of observation and the numbers of children would improve the quality of their findings.

Benjamin et al. [29] compared menus in child care with state dietary regulations and found regulations regarding menus varied from state to state. While this type of research is important for determining whether services are following policy guidelines, the issue of jurisdictional differences may confound results.

A study by Henderson et al. [56] sought to develop a validated tool that could measure nutriton in LDC settings, which included food observation and a scoring system for unhealthy and healthy foods. This study also utilised a menu rating tool and service director interviews. The authors noted the study limitiations as poor generalisabilty, only a single day of observation and potential social desirablity bias.

### Menu review

In 2018, research was published that examined sodium intake from snacks provided in LDC, where meals were photographed pre and post meal times and consumption was estimated. Menus (recipes) were collected for 5 days and evaluated using the Healthy Eating Advisory Service Guidelines (Victoria, Australia) [57]. This research highlighted that recipes from proposed menus differed from actual in service food provision [58], thus, menus can be a poor reflection of actual food provision.

In 2017, a study conducted in New Zealand utilised an online survey tool, email or facsimile to share menus. The results found that most food groups did not meet nutrition guidelines. The study limitations included utilisation of self-reported menus and recipes and found that these did not necessarily reflect food that was provided or consumed [28]. This has been further supported by other research emphasising menu reviews may not reflect actual food provision [30, 31].

A more recent Australian study was designed to assess foods provided by LDC and compared these to the ADG recommendations utilising a review of a two-week menu plan to calculate the number of serves of vegetables, fruit, grains, meat and dairy [15]. Descriptive statistics showed no centre met the recommendations for vegetables, 96% for fruit, 87% for grains, 59% for meat and 89% for dairy [49]. This same study reported that meals differed from what food was actually provided and the focus for this research was on an individual child’s consumption, rather that food provision at a service level.

### Self-reporting

A study by Benjamin-Neelon et al. [48] assessed 13 guidelines that related to restricting or encouraging foods and compared these nutrition practices with voluntary national guidelines for the Australian equivalent of LDC. However, they did not measure food groups or nutrient quantities but whether a food, e.g. a vegetable, was served. This study highlighted significant jurisdictional differences of recommendations in the United States. A limitation of the study, was the reliance on self-reporting food provision by LDC rather than an objective measure of dietary assessment which likely resulted in the overestimation of foods due to a social desirability bias. Further to this a protocol paper by Yoong et al. [59], planned to pilot the effectiveness of an online web based tool, which also relies on self-reporting.

### Food frequency questionnaires (FFQ)/food recalls (FR)

A systematic review [58] was conducted to determine the validity of dietary assessment methods used to determine energy intake in overweight or obese children. This study found that Food Recalls (FR) were repeatedly inaccurate across all age groups and reporters. In addition, a systematic review of the accuracy and reliability of methods used for assessing an individual child’s diet in the school context, found the outcomes were also variable; which supports other research findings to date [60].

Lovell et al. [61] conducted a systematic review on validating Food Frequency Questionnaires (FFQ). This study reviewed 17 articles and found further validation of appropriate population specific tools addressing portion size estimation for young children was required. This review found that FFQ overestimated intakes and individual consumption methods recommended a 3–5 day snapshot of food intake to increase reliability [62]. To the authors knowledge, there has been no systematic review conducted to assess FR/FFQ accuracy and reliability in LDC.

### Portion size

Traditional strategies of measuring food intake can have a high participant burden. To minimise this, the use of portion size assessment tools to estimate individual food consumption has been suggested [37]. This method relies on rigorous training of researchers or service staff and captures an individual’s food provision or consumption. Foster et al. [37], also suggested the nature of the research setting may increase the awareness of the foods being consumed, thus, not accurately representing food provision.

### Plate waste

An older study by Soanes et al. [23], weighed food and deducted the waste to determine individual consumption over three consecutive days and compared this to recommended dietary intakes (RDI) for a range of macro and micro nutrients. This study established that none of the children attending LDC were consuming 50% of their respective RDI whilst attending LDC.

Recently, Seward et al. [63] conducted research on a multi strategy intervention to improve food provision at a service level. Multiple intervention strategies were used to ascertain measures including, aggregated plate waste measures to assess consumption and utilised menus, self-reported by LDC, to determine provision. This study recommended that multiple observation periods be incorporated to better reflect usual child food intake.

There are limitations with these methods. Dietary recording conducted at an individual child level [21] or use of FFQ, often overestimate provision [64] due to social desirability bias [65, 66]. Thompson and Subar [38], confirmed that self-reporting, including FFQ and FR, were problematic as a result of bias for both sample and number of days recorded. The same authors suggest technology [67] should be incorporated to more accurately capture food intake. Further to this, research participants have utilised mobile phones or tablets to capture food images consumed at meal times [68], which can improve accuracy of data collection methods.

In 2014, a pilot study [34], believed to be the first Australian study to examine food provision by food group in LDC services, compared foods provided by a service with 50% of serve recommendations based on the ADG [15]. The authors analysed food and beverages provided to children 2–4 years old from eight LDC services. They weighed all foods and converted them to food groups by a predetermined formula, which converted ingredients into grams of respective food groups. Their analysis showed a significant difference in the number of meat or meat alternatives serves compared to 50% of the ADG (0.33 ± 0.20 serves compared to 0.50 serves; *P* = 0.05). There was no significant difference in the other food groups; however, dairy and fruit exceeded the recommended 50% of serves [34]. This pilot study was conducted to test the suitability of a weighed food data collection method at the service level and found weighing individual ingredient to be an accurate method of data collection.

In summary research highlights international and national inconsistencies when measuring the provision of foods in LDC. Furthermore, at a service level, there is limited research on measurement of food provision. An agreed method of measurement for food provision in LDC services would improve the reporting of food compliance outcomes of services and provide a platform to design resources that can form the basis of consistent recommendations across jurisdictions. A pilot study using an approach considered gold standard for measuring dietary intake in individuals, was deemed a suitable method to accurately determine food provision at a service level [34]. Therefore, this paper builds on that work by providing the methodology for a weighed food data collection method to measure food provision and waste at a service level.

## Methods

### Aim

To propose a suitable method of data collection to accurately report on food provision status of LDC services.

### Study design

A cross-sectional audit in LDC services, in metropolitan Perth, Australia, was conducted by weighing the raw ingredients provided at each meal time; morning tea, lunch and afternoon tea (MT, L, AT), to determine food group provision. This study was approved by the Human Research Ethics Committee (HREC), Edith Cowan University (# 18486).

The data was collected over a three-year period; 2015, 10 services; 2016, 9 services and 2017, 11 services, providing a total sample of 30 services. A required minimum sample size of 27 services was determined using the Wilcoxon signed-rank test at a 5% level of significance and 80% power, with a medium (Cohen’s d = 0.5) effect size. This sample provided sufficient numbers to detect an improvement in the proportion of services (from 40 to 80%) meeting 50% food group compliance based on ADG recommendations [15].

### Participants and recruitment

The sample population included LDC services which operated for more than 8 h per day, 5 days a week and prepared food on site. The services were included if they were located in the Perth, Western Australia metropolitan area (postcodes between 6000 and 6199) and were not previously involved in the pilot study [34]. Services were randomly selected via a publicly available register accessed from Australian Children’s Education and Care Quality Authority (ACECQA) [69], where every 10th centre was telephoned and asked to particpate in the study (maximum follow up attempts 3). If the services met the inclusion criteria, and they agreed to participate, an information letter was sent and followed with a telephone call 2 days later to schedule suitable days for data collection.

### Exclusion criteria

LDC services were excluded from the study if they had participated in the pilot study or in previous years, they declined, or were outside of metropolitan Perth.

### Process of data collection and data entry

**Fig. 1:**
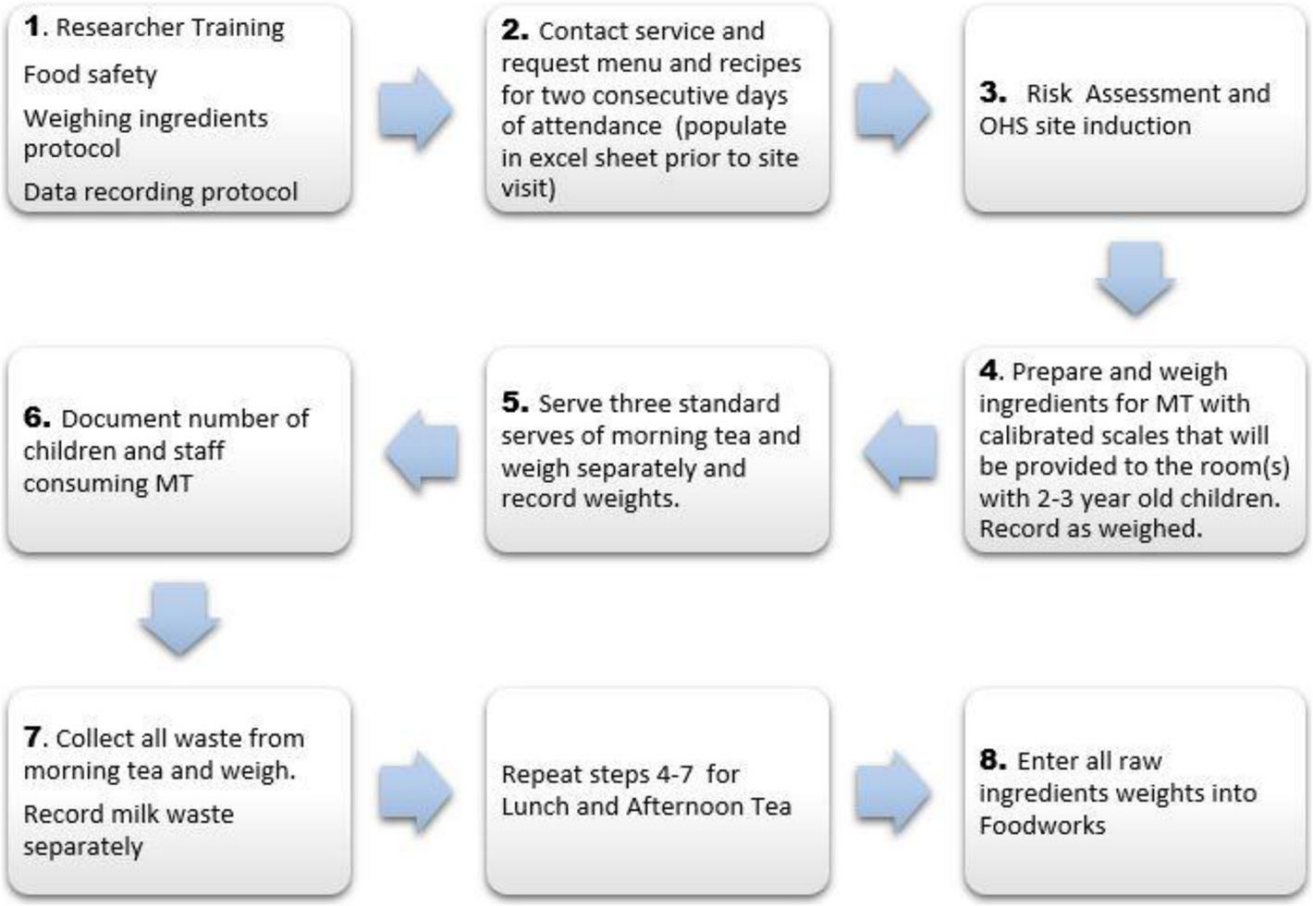
Process of data collection and data entry

Figure 1, represents the preparation and process for data collection and data entry.

#### Research assistant training (Fig. 1, item 1)

Prior to the data collection, research assistants, which included undergraduate Nutrition and postgraduate Nutrition and Dietetics students, underwent 4 h of supervised training [60] to ensure standardised data collection protocol and to become familiar with food preparation in the LDC setting, food measurement using calibrated scales and to familiarise researchers with data recording methods. Training included demonstration and practical application of the major daily tasks outlined in Table 1 below, including training of research assistants to use Foodworks 9, a nutrient analysis Australian software program [70].

**Table 1:** : Summary of major daily tasks, on site, of research assistants

Time	Task
Pre - day 1 or prior to site visit	1. Undertake orientation at facility including policy/procedures and OHS requirements, certificate of currency and risk assessment. 2. Meet with manager/kitchen staff.
Day 1 and 2 (consecutive days): Data Collection	1. Arrive early, prepare and weigh individual morning tea ingredients and record into a pre populated MS Excel spreadsheet. 2. Record children and staff (only if consuming meals) meal attendee numbers 3. Weigh three standard serve sizes - to determine the average meal weight 4. Collect waste from morning tea, weigh and record into a pre populated MS Excel spreadsheet. 5. Prepare and weigh individual lunchtime ingredients and record into a pre populated MS Excel spreadsheet. 6. Record children and staff (only if consuming meals) meal attendee numbers 7. Weigh three standard serve sizes to determine the average meal weight 8. Collect waste from lunchtime, weigh and record into a pre populated MS Excel spreadsheet. 9. Prepare and weigh individual afternoon tea ingredients and record into a pre populated MS Excel spreadsheet. 10. Record children and staff (only if consuming meals) meal attendee numbers 11. Weigh 3 standard serve sizes to determine the average meal weight 12. Collect waste from afternoon tea, weigh and record into a pre populated MS Excel spreadsheet. 13. Repeat this procedure for Day 2.
Day 3	Enter food ingredients and weights into Foodworks

In addition a comprehensive online interactive food safety training package from Environmental Health Australia “I’M ALERT” was completed [71], which included 23 sections on basic food safety principles.

#### Contact service and request menu and recipes (Fig. 1, item 2)

Research assistants contacted the LDC 2 weeks prior to scheduled site visit to request the menu and recipes that would be prepared on two consecutive data collection days. These recipes were pre-populated into an Excel spreadsheet including the number of anticipated serves. On the day of data collection, the actual weight of the recipe ingredients and number of attendees were documented in a separate column for comparison at a later date.

#### Risk assessment and induction (Fig. 1, item 3)

Prior to data collection days, Occupational Health and Safety (OHS) induction was completed. This process included familiarisation of the centre, emergency procedures, and completion of risk management forms and exchange of certificate of currency for insurance.

#### Preparation and weighing raw ingredients (Fig. 1, item 4)

Day 1 and 2: The raw ingredients used to prepare MT, L and AT meals were weighed with calibrated scales (A&D Australasia, SJ-5001HS) and recorded over two consecutive days. As most services have multiple rooms, the weight of the ingredients had to reflect proportionately what was offered to each room. The weight, in grams, of all ingredients provided, for each room, were entered into the pre-populated Microsoft Excel sheet in the “actual” food weight column. It was deemed by the chief investigators that data gathering for all age groups would allow for more accurate reflection of provision for children 2–3 years at a later date if required.

Day 3: Each raw ingredient weight was added for each recipe, where there were multiple recipes for a meal occasion, these were added separately as was milk (full cream unless otherwise specified), into Foodworks [70]. The number of children and staff that were consuming food at each meal time was also added for each recipe to Foodworks. The recipes were used to populate a daily meal plan for a reference child: 2–3 year old boy, including; MT, L and AT. A 2–3 year old boy was chosen as a reference child in Foodworks as the highest proportion (approx. 45%) of children attending LDC are aged 2–3 years [72] and for children aged 2–3 years there is no gender difference between food group serve size recommendations [36].

Where product items were unknown or not recognised by Foodworks a standard equivalent item was chosen, to reduce discrepancies in data collation.

Foodworks [70] data was exported into Microsoft Access [73] and then into IBM SPSS Statistics [74]. Food group and nutrient line data, by child, by day, by service, were available for analysis.

Included in the analysis was discretionary food, which is described as foods and drinks that do not fit into the Five Food Groups because they are not considered a part of a healthy diet as they are too high in saturated fat and/or added sugars, added salt or alcohol and low in fibre [75]. Where discretionary foods were identified the individual ingredient weights were converted to a serve based on the Australian Dietary Guideline recommendations (one serve of discretionary food =600 kJ) [75]. The discretionary food serves per meal were added separately to the final SPSS data file for each meal time as this was not captured by Foodworks. Further to, the fat allowance was based on the ADG [15]. In addition, Nutrient Reference Values [76], provided guidelines for fat as a percentage of energy intake and average intake allowances for a variety of fatty acids for comparative analysis.

A one-sample t-test or Wilcoxon signed-rank test was used to compare food provision to the 50% recommended serves of each food category, discretionary serve and fat allowance for children aged 2–3 years (Table 2) [36]. *P* value ≤0.05 was assumed statistically significant.

**Table 2:** 50% of recommended serves of food groups, fat allowance and discretionary foods, for 2–3 year olds based on ADG [15]

Food category	Recommended serves for 2–3 year olds [15]	50% Recommended serves
Vegetables	2.5	1.25
Fruit	1	0.50
Grains/cereals	4	2.00
Milk/milk alternatives	1.5	0.75
Lean meat/meat alternatives	1	0.50
Fat allowance	35–40% of TEI ^a^	35–40% of TEI ^a^
Discretionary foods	0.00 ^b^	0.00 ^b^

^a^Fat allowance based on 35–40% of Total Energy Intake (TEI) [35]

^b^Eat For Health recommends that discretionary foods should not be offered in ECEC services [75]

Descriptive statistics were be utilised to describe the data between the two consecutive days, the average of data collection and between the services for each food category. Shapiro-Wilk test will be used to verify normality.

#### Weigh three standard serves of meal time and record weights (Fig. 1, item 5)

Kitchen staff/educators were asked to serve up three standard meals that would represent a typical serving offered to the room, three standard plates were tared. Meal weights were documented then averaged. This average weight was recorded as a standard serve for the specified meal time. The standard serve was used to calculate proportion of ingredients of cooked weight at a later date if required, particularly where leftovers were recorded. Leftovers were considered as any food that was held back to be served at a later stage that would affect the total weight of food that had been weighed for a specific meal time. Leftovers that are part of the original total weight of ingredients are weighed and this weight is documented minus storage containers, to give a total weight of ingredients that can be deducted from a specific meal time.

Research assistants are required to document the volume of milk that was allocated to the room and then document the volume of milk that is returned and will be offered later at a different meal time. The total milk minus the volume of milk that will be used at a later stage will equal the figure entered into Foodworks as provision, milk waste should be added as per Fig. 1, item 7.

### Document number of children and staff consuming MT (Fig. 1, item 6)

The number of children and staff sharing the meal in the room for each specific meal time (MT, L and AT) are counted, documented and utilised for food group provision calculations for each meal time.

#### Measuring waste (Fig. 1, item 7)

Aggregated waste was measured for each meal time excluding leftovers, following the steps below;

1. Place a plastic bag inside a bucket and record the weight.
2. Scrape all food from plates from the relevant room, into the bucket and record weight, check floors and tables for any additional waste for inclusion.
3. Weight of leftovers (measure milk separately) are recorded separately.
4. Repeat for each mealtime.
5. At the end of the day, reweigh the bucket containing the scraps and record second weight, average and enter into the excel sheet.
6. Milk waste should be collected in a jug (tared) to measure total waste for each meal time and recorded separately to food waste weight.

### Reliability

Reliability of the data collection process relates to the consistency in training of all research assistants prior to data collection ensuring adherence to the protocol thus, quality of data. As this data collection method measured actual weight of food ingredients there was limited opportunity for variability of actual weights and social desirability bias. A second set of calibrated scales were available if required. Validity could have been increased if raw ingredients were re-weighed three times and an average taken. This was not practical due to time constraints around meal preparation in LDC service kitchens. Reliability of analysis was increased by entering all data into same version of Foodworks.

### Strengths and limitations

- Training of researchers reduces the variability of data collection and is a critical part of the process.
- A weighed food ingredient measure provides an accurate representation of food provided at a service level.
- This method is less onerous on individuals and services, which increases the level of willingness to participate in the study.
- Data is collected across two consecutive days, increasing the number of days would increase the transferability of the outcomes.
- Services were also aware they were participating in research and may potentially supplement/alter ingredients, which could skew findings due to social desirability bias.

## Discussion

The method of data collection is critical to accurately measure food provision in LDC. The variation in research methods limits comparability of food provision over time, yet historically these research projects are used to determine and compare compliance of food provision in LDC. Food weight records are deemed gold standard when determining individual food consumption [32, 39], yet there is a paucity of evidence for accurately measuring food provision at a service level in LDC. Dietary assessment pertaining to weighed food records were adapted for a service setting and were more accurately able to reflect food group provision. We recommend that the measurement of LDC food provision data, be captured using this method in order to more effectively determine change, compare studies over time and report on the current status of food provision at a service level which could be extended to a national bi-annual audit in LDC. An audit would enable food provision surveillance at a service, state and national level and could guide intervention strategies and resource development in a more targeted way. The audit could be coordinated collaboratively between an accrediting body, such as ACECQA and the tertiary sector, with specific oversight from early years nutrition specialists such as members of the National Nutrition Network-Early Childhood Education and Care (NNN-ECEC), whose mission is to promote best practice provision of food within ECEC services to facilitate positive short and long term nutrition, health and development outcomes for children who attend care.

## Data Availability

This paper is a study protocol hence no linked data.

## Abbreviations

ADG: Australian dietary guidelines
AT: Afternoon tea
ECEC: Early childhood education and care
FFQ: Food frequency questionnaire
FR: Food recall
L: Lunch
LDC: Long daycare
MT: Morning tea
